# The grain quality of wheat wild relatives in the evolutionary context

**DOI:** 10.1007/s00122-021-04013-8

**Published:** 2021-12-17

**Authors:** Frederike Zeibig, Benjamin Kilian, Michael Frei

**Affiliations:** 1 Department of Agronomy and Crop Physiology, Institute of Agronomy and Plant Breeding I, Justus-Liebig-University, 35392 Giessen, Germany; 2 Global Crop Diversity Trust, 53113 Bonn, Germany

## Abstract

**Key message:**

We evaluated the potential of wheat wild relatives for the improvement in grain quality characteristics including micronutrients (Fe, Zn) and gluten and identified diploid wheats and the timopheevii lineage as the most promising resources.

**Abstract:**

Domestication enabled the advancement of civilization through modification of plants according to human requirements. Continuous selection and cultivation of domesticated plants induced genetic bottlenecks. However, ancient diversity has been conserved in crop wild relatives. Wheat (*Triticum aestivum* L.; *Triticum durum* Desf.) is one of the most important staple foods and was among the first domesticated crop species. Its evolutionary diversity includes diploid, tetraploid and hexaploid species from the *Triticum* and *Aegilops* taxa and different genomes, generating an AA, BBAA/GGAA and BBAADD/GGAAA^m^A^m^ genepool, respectively. Breeding and improvement in wheat altered its grain quality. In this review, we identified evolutionary patterns and the potential of wheat wild relatives for quality improvement regarding the micronutrients Iron (Fe) and Zinc (Zn), the gluten storage proteins α-gliadins and high molecular weight glutenin subunits (HMW-GS), and the secondary metabolite phenolics. Generally, the timopheevii lineage has been neglected to date regarding grain quality studies. Thus, the timopheevii lineage should be subject to grain quality research to explore the full diversity of the wheat gene pool.

## Introduction

Wheat is one of the world’s most important crops. It provides 19% of human calorie intake and 21% of protein intake (Shiferaw et al. 2013). Hexaploid bread wheat (*Triticum aestivum* L., 2*n* = 6*x* = 42, BBA^u^A^u^DD) accounts for about 90% of wheat production, and tetraploid pasta wheat (*Triticum* *durum* Desf., 2*n* = 4*x* = 28, BBA^u^A^u^) is the 10th most important staple crop (International Grains Council [IGC] (2020). World Grain Statistics 2016). The two economically most important wheat species have different levels of ploidy. This is due to the evolutionary history, in which the process of domestication plays a predominant role (Dubcovsky and Dvorak 2007). Domesticated plants fulfilled the requirements of human cultivation and dietary preference. The main characteristic of domesticated cereals is the loss of the ability to survive independently in the wild (Meyer and Purugganan 2013; Purugganan and Fuller 2009; Zohary et al. 2012). The center of wheat diversity and initial domestication lies in the Fertile Crescent. The evolutionary history of wheat is characterized by the allo-polyploidization of the originally diploid *Triticum* and *Aegilops* species, which led to the emergence of tetra- and hexaploid wheat species. Human cultivation of the tetraploid wild emmer *Triticum dicoccoides* (Körn. ex. Aschers. & Graebner) Schweinf. (2*n* = 4*x *= 28, BBA^u^A^u^) resulted in the emergence of the domesticated *Triticum dicoccon* Schübl. (2*n* = 4*x* = 28, BBA^u^A^u^) and later *T. durum*. The domesticated tetraploid species as well as *T. aestivum*, of which no direct hexaploid wild relative has yet been identified, expanded their cultivation area by human migration and trade (Kilian et al. 2010; Zohary et al. 2012). Migration from their center of diversity and continuous breeding efforts reduced the genetic diversity of *T.* *aestivum* and *T. durum* (Tanksley and McCouch 1997; Ross-Ibarra et al. 2007). Currently, climatic conditions are changing, posing a threat to the productivity of the cultivated wheats (Zhao et al. 2017; Iizumi et al. 2021; Trnka et al. 2014). The wild and domesticated relatives of wheat are a valuable source of beneficial genetic diversity that can be used for crop improvement (Dempewolf et al. 2014; Henry and Nevo 2014; Brozynska et al. 2016; Kilian et al. 2021; Sharma et al. 2021). Wild relatives of wheat are well adapted to their natural habitats, which can be harsh, so they carry useful traits for abiotic stress tolerance (Henry and Nevo 2014; Singh et al. 2012). Their genetic diversity has also been exploited for their resistance to various pests, e.g., hessian fly (Nsarellah et al. 2003) and diseases, such as powdery mildew (Li et al. 2020a) and leaf rust (Fatima et al. 2020; Narang et al. 2020). These beneficial traits can be incorporated into modern wheat cultivars via (pre-)breeding programs (Kilian et al. 2011, 2021; Sharma et al. 2021) and thus illustrate the value of this genepool for securing the future cultivation of wheat. In order to harness and conserve the beneficial diversity of wild relatives, genebanks play an important role as ex situ conversation sites (Dempewolf et al. 2014; Kilian et al. 2021). An overview of ex situ *Triticum* collections can be found in Sharma et al. 2021.

Due to wheat’s role as a staple, the quality of the grain is of great importance. Its daily consumption in many parts of the world makes it a major source for calorie intake, but it also provides micronutrients and vitamins. Breeding for energy yield and grain quality for purposes such as baking has long been a major goal in wheat breeding and enabled food security for a growing population. However, the focus on micronutrients and allergenicity has only been a recent trend in wheat breeding. Especially in less diversified diets, a low concentration of micronutrients in wheat appears to be a major problem. This leads to micronutrient deficiencies (“hidden hunger”), especially for the crucial micronutrients iron (Fe) and zinc (Zn) (Graham et al. 2001, 2007; Johns and Eyzaguirre 2007). The main symptom of Fe deficiency is anemia, and Zn deficiency can affect growth and development (Prasad et al. 1961; Stein 2010; Gibson et al. 2010). To combat hidden hunger, one commonly used approach is biofortification (= enrichment of micronutrients) of crops (Johns and Eyzaguirre 2007). Crop biofortification can be achieved through agronomic strategies such as foliar fertilization or via various breeding methods such as introgression. Some wild relatives of wheat accumulate micronutrients very efficiently in the grain and offer high genetic diversity for this trait (Arora et al. 2019; Cakmak et al. 2000; Chhuneja et al. 2006; Chatzav et al. 2010). Therefore, they can be used as a breeding resource for biofortified crops (Cakmak et al. 2004; Rawat et al. 2009).

Wheat is consumed in various forms, for example as bread, pasta, bulgur and couscous. An important quality-determining characteristic is gluten, which comprises a group of seed storage proteins composed of glutenins and gliadins. While the former is very important for dough quality and elasticity, the latter is responsible for viscosity (Wieser 2007; Shewry 2019). However, gliadins can trigger an autoimmune disease called celiac disease (CD) (Biesiekierski 2017). Currently, the only strategy for CD patients is to avoid wheat products. The wheat genepool may harbor diversity that can help to combine improved dough quality properties of glutenin with less toxic and non-immunogenic gliadin variants for CD-safe wheat.

Phenolics are bioactive components in the grain with promising health benefits for the consumer (Laddomada et al. 2015). Phenolic compounds can be grouped into phenolic acids, flavonoids, stilbenes, coumarines, lignans and tannins. Phenolic acids are the most abundant component in wheat and are predominantly stored in the aleurone layer and the bran (Liyana-Pathirana and Shahidi 2006; Laddomada et al. 2015). The domesticated einkorn and emmer were assumed to be enriched in phenolic acid content (Serpen et al. 2008; Barański et al. 2020), and thus, wild wheats could be a valuable source for the enrichment of phenolic acids in the whole wheat grain.

This review provides an overview of the state of research on grain quality traits in the wheat genepool, including micronutrients, gluten and phenolics. Research on grain quality parameters in wheat relatives is summarized and assessed in the context of evolutionary history.

## The process of domestication

Humans selected wild plants with favorable characteristics for cultivation and preparation to ensure food security. This process genetically transformed the plants into a domesticated plant that is dependent on human care and thus loses its ability to survive in the wild (Doebley et al. 2006). The modified plants showed a distinctive morphology that is referred to as the domestication syndrome (Hammer 1984). The domestication syndrome evolved from the selection of wild plants for higher germination rates and easier harvesting (Purugganan and Fuller 2009). The most common traits are the loss of seed shattering, increased seed size and alteration of the reproductive organs (Doebley et al. 2006; Ross-Ibarra et al. 2007; Meyer and Purugganan 2013). These domestication traits evolved through a combination of conscious and unconscious selection. Humans actively carried out conscious selection by selecting a favored trait and thus forwarded this trait into the next generation. Unconscious selection, on the other hand, favored the adaptability to the new agro-ecological environment shaped by human farming systems. As a result, the plant improved its performance in the field and was chosen for further breeding (Zohary 2004). In the beginning, wild and cultivated plants grew close to each other, which led to the exchange of genes via cross-pollination and gene flow (Gross and Olsen 2010; Przewieslik-Allen et al. 2021; He et al. 2019). This scenario still occurs today in the centers of origin of crops. However, human intervention has morphologically and genetically modified the wild plants, resulting in a newly domesticated species (Kantar et al. 2017). In addition to selection for domestication traits, selection for plant improvement further differentiated the domesticated species. This process is called diversification phase and involves the adaption of plants to new environments and selection for quality traits that differed individually for each crop (Meyer and Purugganan 2013). As a result, the differently pronounced selection pressure led to further divergence within the species. Genetic alteration and introduction into new habitats led to adaptation of a domesticated species. In summary, the domestication and improvement phase increased diversity in terms of the number of newly emerged domesticated taxa. However, it is important to focus on the initial situation of domestication. The onset of domestication occurred independently in different areas around the world at different times, but the traits of interest were similar (Purugganan and Fuller 2009; Meyer and Purugganan 2013). From the broad wild diversity that was available to humans, only a few plants were selected and used as founder crops (Ladizinsky 1985). Consistent cultivation and selection within these founder crops led to a genetic bottleneck that excluded many wild alleles from the domestication process (Doebley et al. 2006; Stetter et al. 2017). The diversity retrieved during the domestication and improvement phase cannot compensate for the reduced diversity at the beginning (Smýkal et al. 2018). The founder crop (population) experienced high selection pressure on domestication traits. Increased selection led to selective sweeps that are reflected in a reduced diversity of domestication loci compared to other loci (Smýkal et al. 2018; Maccaferri et al. 2019; Pont et al. 2019). Most early domesticates were annuals because the fixation of traits through an annual selection cycle was more successful and faster compared to perennials. Thus, selection pressure on desired traits accelerated the loss of diversity in the corresponding loci and reduced overall diversity (Maccaferri et al. 2019).

## Domestication history of wheat and its resulting diversity

Wheat’s allo-polyploidy accelerated its wider distribution, as polyploid species show a better adaption rate to new environments and outperform their ancestors with a lower degree of ploidy in agronomic traits (Dubcovsky and Dvorak 2007). Therefore, farmers favored them, which led to a larger cultivation area and facilitated their spread from the Fertile Crescent across the world.

### Diploid wheats

**Fig. 1:**
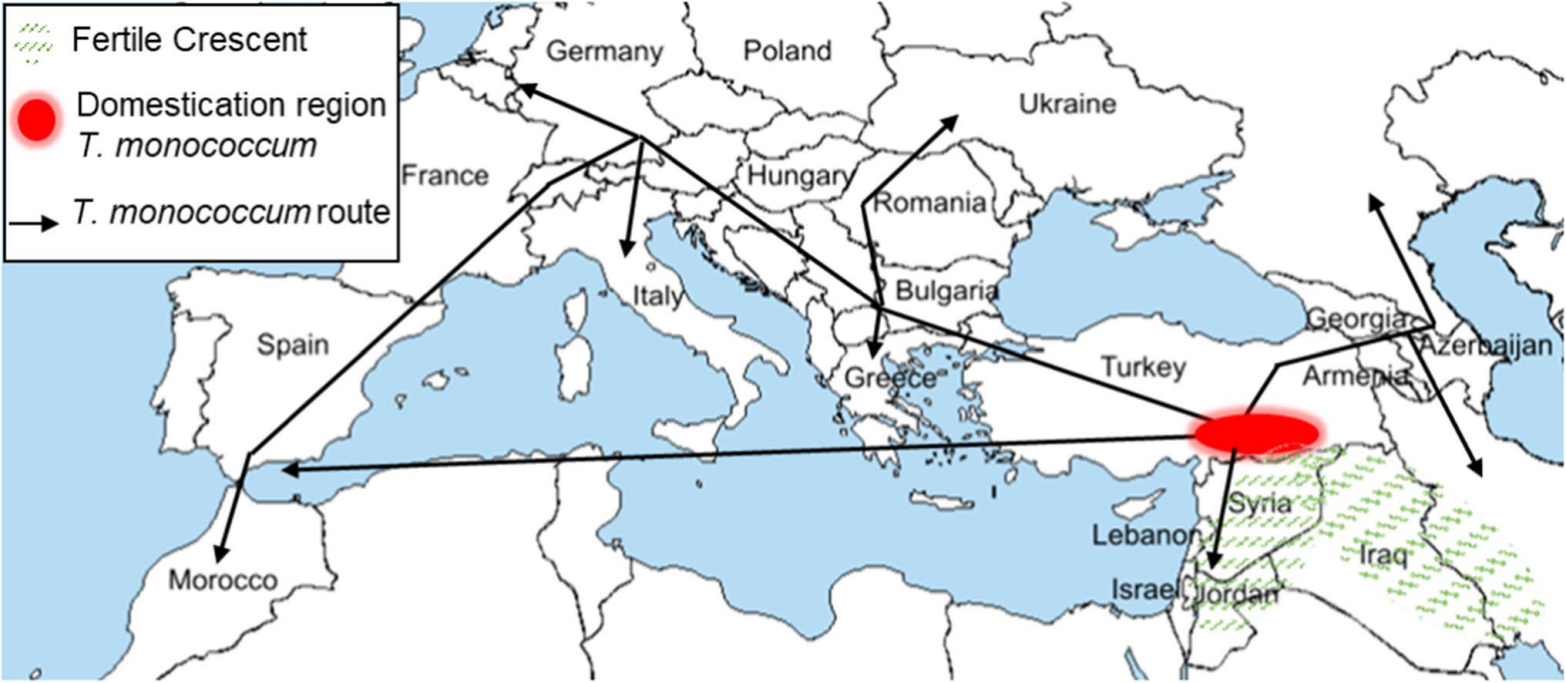
Origin and spread of domesticated einkorn wheat *T. monococcum*. Green dashed fields represent the Fertile Crescent; red circle is the domestication region of *T. monococcum (*Heun 1997; Kilian et al. 2007b); arrows indicate T*. monococcum*’s dispersal pathways (Zaharieva and Monneveux 2014; Brandolini et al. 2016)

Diploid wheats (AA) belong to the oldest cultivated plant species. Wild representatives of the diploid wheat group are *Triticum boeoticum* Boiss (2n = 2x = 14, AA) and *Triticum urartu* Thumanjan ex Gandilyan (2n = 2x = 14, A^u^A^u^). *Triticum monococcum* L. (2n = 2x = 14, AA) is the domesticate of *T.* *boeoticum*. Together, they form the einkorn group of wheat. The Karacadağ and Kartal-Karadağ mountains in Turkey have been identified as their center of domestication (Heun 1997; Kilian et al. 2007b). From here, einkorn migrated east (Armenia, Georgia, Iran) and west via the Bosporus (Greece and toward Central Europe). Another route involved maritime transport to the Maghreb and the Iberian Peninsula. These routes are supported by archaeological evidence, but also the population structure of the einkorn genepool that was shaped by the migration routes (reviewed in: Zaharieva and Monneveux 2014; Brandolini et al. 2016) (Fig. 1).

*T. urartu* is phenotypically similar to *T. boeoticum* (Morrison 1993) but can be distinguished genetically (Yildirim and Akkaya 2006; Konovalov et al. 2010). *T.* *urartu* represents the A genome donor of tetra- and hexaploid wheats (Huang et al. 2002b; Yildirim and Akkaya 2006). *T. urartu* populations occur in the Fertile Crescent and are found in Lebanon, Turkey, Iraq, Iran and Transcaucasia. This geographic distribution also shaped the genetic diversity of *T. urartu* by forming groups according to bioclimatic and topographic criteria (Brunazzi et al. 2018).

### Tetraploid wheats

Hybridization between *T.* *urartu* and a close relative of *Aegilops* *speltoides* Tausch (2n = 2x = 14, BB/GG), a member of the *Sitopsis* section in *Aegilops*, initiated the formation of the tetraploid wild wheat, *T.* *dicoccoides*. This wild emmer wheat further evolved into the domesticated *T. dicoccon*. However, the emmer lineage is not the only tetraploid wild wheat. *Triticum araraticum* Jakubz. (2n = 4x = 28, GGA^u^A^u^) is also a descendant of an independent hybridization between *T.* *urartu* and *Ae. speltoides*. Although both tetraploid wheat lineages share potentially the same parental species, they differ in the structure of the genome and time of origin (Adonina et al. 2015). Diversity analysis clearly showed a shared ancestry of *Ae.* *speltoides* and tetra- and hexaploid wheats. *Ae.* *speltoides* shared nuclear and cytoplasmic loci with *T.* *dicoccoides* and *T.* *araraticum*, which differed between the two wild tetraploid wheat species. Therefore, it was proposed that both genomes were derived (at least in parts) from *Ae.* *speltoides* as a maternal donor (Kilian et al. 2007a; Golovnina et al. 2007). However, the G genome of *T.* *araraticum* shows a closer relationship with *Ae. speltoides*, while the B genome donor is more distant to *Ae. speltoides* (Huang et al. 2002b; El Baidouri et al. 2017; Bernhardt et al. 2017).

### Domestication and cultivation of the BBAA lineage and the hexaploid BBAADD

**Fig. 2:**
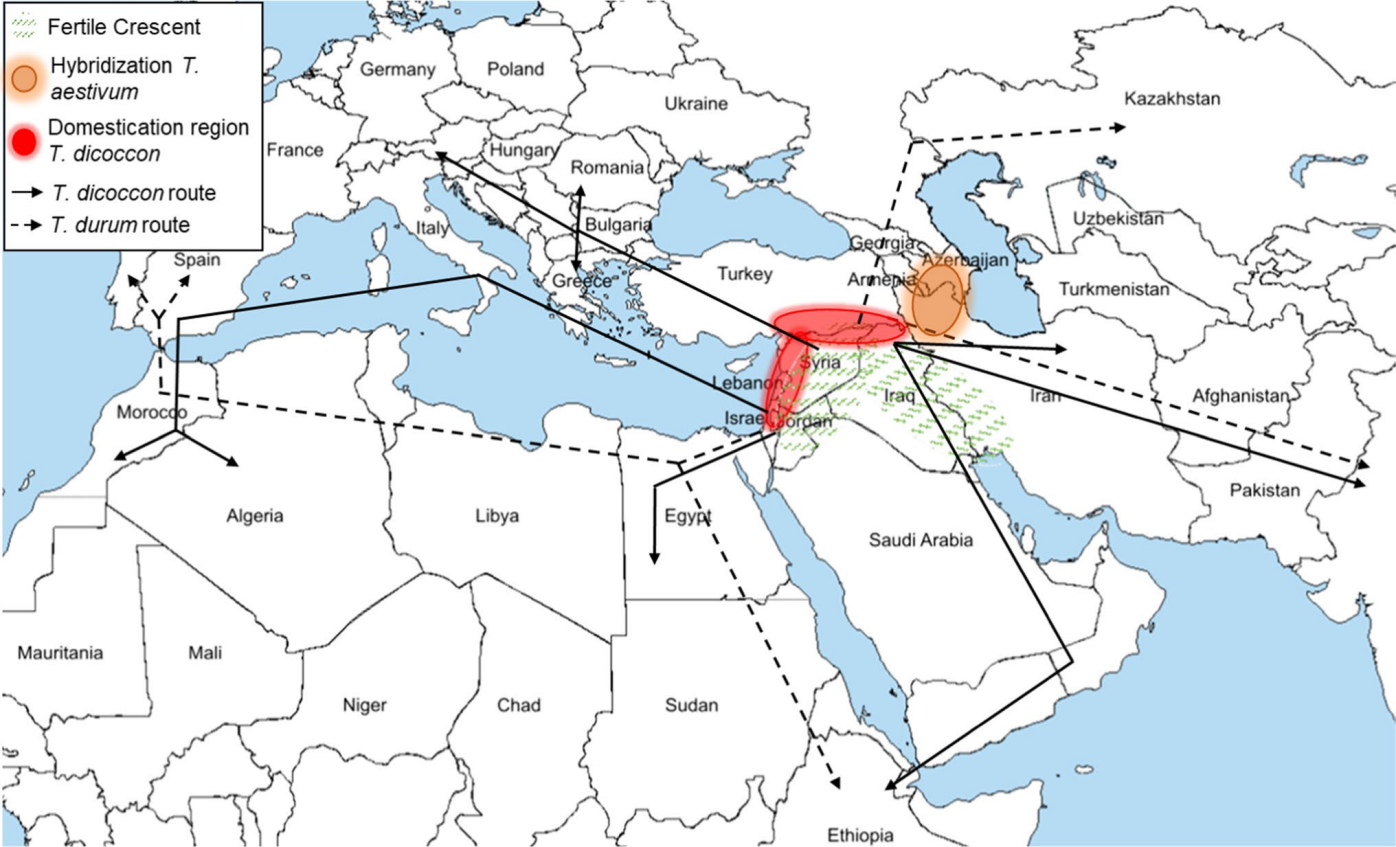
Origin and spread of domesticated emmer and durum wheat. Green dashed fields are the Fertile Crescent; red circles are the domestication regions of *T. dicoccon* (Oliveira et al. 2020); solid arrows indicate dispersal routes of *T. dicoccon* (Martínez-Moreno et al. 2020; Badaeva et al. 2015); dashed arrows indicate dispersal route of *T. durum* (Moragues et al. 2007; Kabbaj et al. 2017; Martínez-Moreno et al. 2020); orange circle is the potential hybridization site of *T. aestivum* (Petersen et al. 2006; Salamini et al. 2002; Huang et al. 2002a; Pont et al. 2019)

Molecular analysis of *T. dicoccoides* revealed two subgroups with distinct geographic origin. The southern Levant subgroup included genotypes from Israel, Palestine, Jordan, Syria and Lebanon. Accessions belonging to the other group were associated with the Central Eastern Fertile Crescent extending from Turkey to Iraq and Iran (Luo et al. 2007; Ozkan et al. 2005; Maccaferri et al. 2019). Some studies suggested a monophyletic origin of domesticated emmer from within the Central Eastern group. However, archaeological data indicate early cultivation of emmer in the Southern Levant (reviewed in: Özkan et al. 2011). Recent analysis suggested a multi-regional origin by showing that domesticated emmer contains alleles from both *T. dicoccoides* populations. It was concluded that wild emmer was pre-cultivated in the southern Levant and met wild emmer from the Central Eastern group through migration northwards, where it was eventually domesticated (Oliveira et al. 2020). *T. dicoccon* spread in all directions via various routes. Europe was reached via the Bosporus, the Balkans and the Iberian Peninsula. Africa was reached via the southern Levant and the Arabian Peninsula, and the spread to Asia was via Iran (reviewed in: Martínez-Moreno et al. 2020; Badaeva et al. 2015) (Fig. 2).

*T. durum* is currently the economically most important cultivated species from the tetraploid BBAA genepool. Durum wheat most likely originated in the eastern Mediterranean region (Luo et al. 2007; Kabbaj et al. 2017), which is supported by a high genetic diversity in durum wheat accessions from the Central Fertile Crescent (Baloch et al. 2017). However, its evolution has not yet been fully elucidated. Phylogenetic analysis of wild and domesticated emmer, durum wheat landraces and durum cultivars revealed a close relationship between durum wheat landraces and domesticated emmer from the southern Levant. This suggests a rather linear evolution, in which increased selection and improvement on *T. dicoccon* facilitated the emergence of *T. durum* landraces. Moreover, modern durum wheat cultivars showed the closest relationship to durum wheat landraces from North Africa and Transcaucasia (Maccaferri et al. 2019). In contrast, exome sequencing data of the wheat genepool identified *T.* *dicoccoides* as a direct donor to *T. dicoccon* and *T. durum*, suggesting an entangled evolution (Pont et al. 2019). From its potential center of origin, durum wheat spread widely following similar expansion routes as *T.* *dicoccon* (Moragues et al. 2007; Kabbaj et al. 2017; Martínez-Moreno et al. 2020) (Fig. 2).

The emmer lineage evolved further via another hybridization event, which gave rise to the hexaploid wheats. Most likely, domesticated tetraploid wheat and *Aegilops* *tauschii* Cosson (2n = 2x = 14, DD) hybridized in the area of northeastern Iran and the southwestern Caspian Sea, giving rise to the hexaploid bread wheat, *T. aestivum* (Petersen et al. 2006; Salamini et al. 2002; Huang et al. 2002a; Pont et al. 2019). The highest genetic diversity for hexaploid bread wheat was found in the Near and Middle East, which is probably the center of diversity (Huang et al. 2002a). Hexaploid bread wheat is based on a founder race that further evolved into two hexaploid wheat genepools (Pont et al. 2019). Moreover, the wheat population can be divided into a western and far eastern subpopulation, the latter resembling the ancient wheat due to less introgression from *Ae. tauschii* (Wang et al. 2013). Polyploidization limited the initial diversity of the new species, as only a small number of genotypes were potentially involved in its emergence (Dubcovsky and Dvorak 2007; Pont et al. 2019). However, due to introgression from *T. dicoccoides* into the hexaploid wheat genome, some diversity was retained (He et al. 2019).

### Domestication and cultivation of the wheat G genome containing lineage

**Fig. 3:**
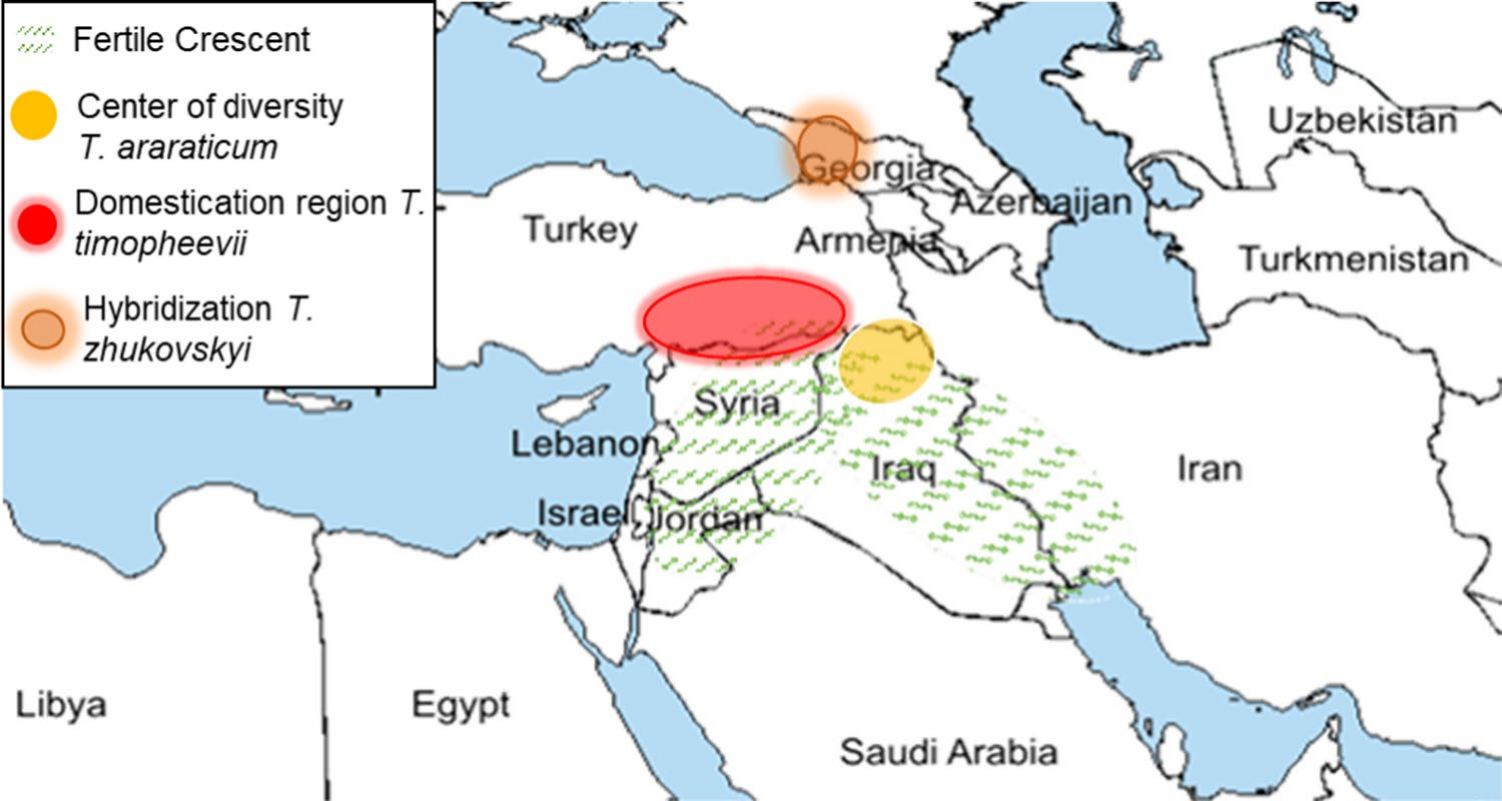
Origin and spread of GGAA wheat. Green dashed fields are the Fertile Crescent; yellow circle indicates the center of diversity of *T.* *araraticum* (Gornicki et al. 2014; Badaeva et al. 2021); red circle is the domestication region of *T. timopheevii* (Mori et al. 2009; Badaeva et al. 2021); orange circle indicates the hybridization site of *T.* *zhukovskyi* (Badaeva et al. 1994; Badaeva et al. 2016; Badaeva et al. 2021)

In contrast to the emmer lineage, the timopheevii lineage with the genomic constitution GGAA received less attention. The evolutionary history of the GGAA wheat genepool is complex, as several chromosomal rearrangements were involved (Badaeva et al. 2021). The wild ancestral form of the timopheevii lineage is *T.* *araraticum*. Northern Iraq is the center of diversity and origin of *T. araraticum* (Gornicki et al. 2014; Badaeva et al. 2021) (Fig. 3). *T.* *araraticum* comprises two subgroups. One subgroup (ARA-0) is widespread, while the other (ARA-1) was only found in south-eastern Turkey and north-western Syria (Badaeva et al. 2021).

*Triticum timopheevii* (Zhuk.) Zhuk (2x = 4n = 28, GGA^u^A^u^) is the domesticate of *T. araraticum*. The origin of *T. timopheevii s.str.* (found in Georgia) remains unclear, but was probably introduced from Turkey (Badaeva et al. 2021). Badaeva et al. (2021) also discuss the potential sister-group relationship between the Georgian *T. timopheevii s.str.* and the prehistoric and widespread *T. timopheevii s.l*. (‘New Glume Wheat’), with the oldest known records being of Turkish origin.

The mixed cultivation of *T.* *monococcum* and *T. timopheevii* in western Georgia facilitated hybridization between the two. This event resulted in the hexaploid *Triticum zhukovskyi* Men. & Er*.* (2*n* = 6*x* = 42, GGA^u^A^u^A^m^A^m^) (Badaeva et al*.*, 1994; Badaeva et al*.*, 2016; Badaeva et al., 2021) (Fig. 3). However, Pont et al. (2019) suggested a hybridization between *T. araraticum* and *T. boeoticum* for the formation of *T. zhukovskyi*.

## Micronutrients in wheat wild relatives

Iron (Fe) and Zinc (Zn) deficiency are major forms of micronutrient disorders in human diets relying on one major crop. Fe deficiency leads to anemia and insufficient Zn uptake impairs various essential functions of the body including growth, development and the immune system (Stein 2010). The large wheat genepool described earlier may harbor beneficial genetic variation for an increased accumulation of micronutrients in the edible parts of the wheat crop. Furthermore, the evolutionary pattern can be used to identify trends and underutilized species for biofortification.

### Wheat wild relatives show high enrichment and variation for Fe and Zn in the grain

*Aegilops* species and the einkorn and emmer lineage were of interest for the grain micronutrient concentration and the variation. The wild species harbored a high variation for Zn grain concentration, whereas *Aegilops* species showed elevated Fe grain concentration.

**Fig. 4:**
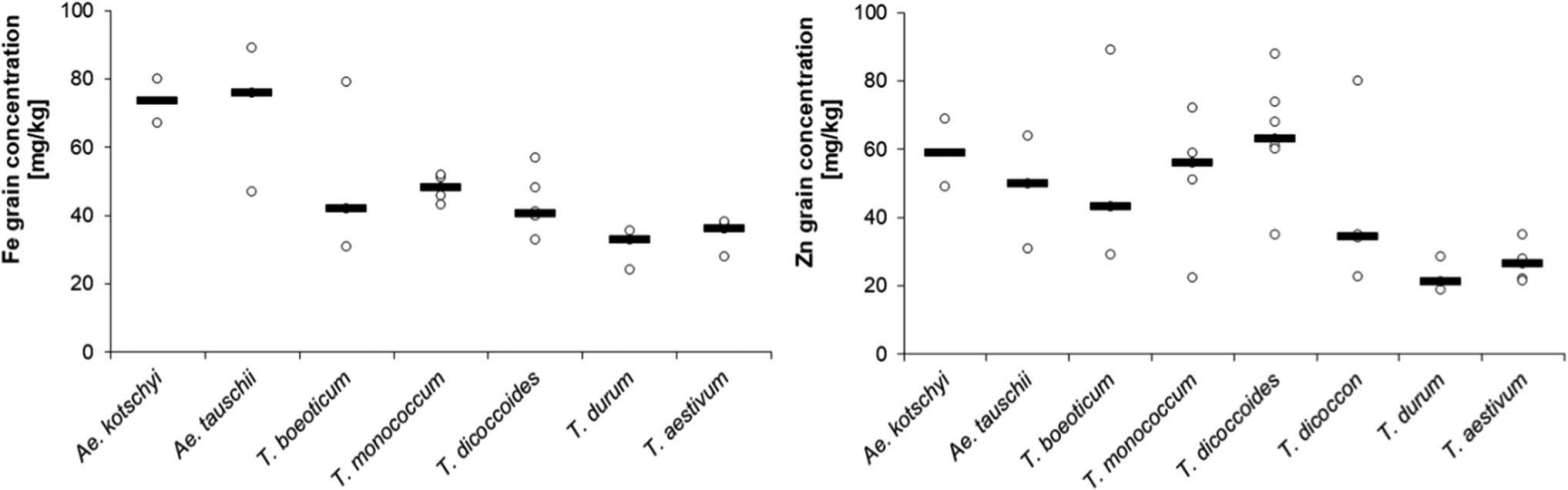
Distribution of mean Fe or Zn grain concentration measured in different studies for different species. The bold lines are the median of different species means. Dots represent individual mean values. Excel script from Weissgerber et al. (2015)

Domesticated einkorn wheat forms a valuable source for biofortification, especially for Zn (Table 1) (Ozkan et al. 2007; Erba et al. 2011). In addition, high values of Fe and Zn were consistently found in *T.* *monococcum* accessions across different locations (Ozkan et al. 2007; Erba et al. 2011). Therefore, an underlying genetic component is likely. Compared to *T. monococcum*, the variation in Zn and Fe concentrations was higher in *T.* *boeoticum* in the studies comparing the wild and domesticated forms (Fig. 4, Table 1). This underlines the greater potential for genetic variation in crop wild relatives. Furthermore, grains of *T. zhukovskyi* could be potentially enriched in Zn, due to the fact that it contains the A genome of *T. monococcum*. Among the few studies available, most focused on *T.* *monococcum* and *T.* *boeoticum* and excluded *T.* *urartu*, the actual A genome donor of emmer, durum, bread wheat and timopheevii wheat. Because of this ancestry, exploring the micronutrient content and accumulation of *T. urartu* might be valuable for the biofortification of its descendants. Diploid wheats have been less extensively investigated than their tetraploid relatives from the emmer lineage. One reason for this lack of interest could be their inferior agricultural performance or the challenges in introgressing beneficial alleles into bread wheat. Thus, there is still a research gap that needs to be explored.

**Table 1:** Summary of studies in which more than one genotype of the respective species was examined for Fe and Zn concentration in the grain

	Fe					Zn				
Species	Mean (mg/kg)	Range (mg/kg)	Difference (mg/kg)	Number of Studies	References	Mean (mg/kg)	Range (mg/kg)	Difference (mg/kg)	Number of Studies	References
*Ae. cylindrica*	67	52–93	41	1	5	39	32–52	20	1	5
*Ae. geniculata*	70	52–82	30	1	5	38	32–41	9	1	5
*Ae. kotschyi*	74	23–91	68	2	4,5	59	22–59	37	2	4,5
*Ae. longissima*	73	60–82	22	1	5	42	25–51	26	1	5
*Ae. peregrina*	53	34–82	48	1	5	40	33–49	16	1	5
*Ae. tauschii*	71	30–99	69	3	2,4,9	48	18–69	51	3	2,4,9
*Ae. ventricosa*	66	55–94	39	1	5	34	24–39	15	1	5
*T. aestivum*	35	21–51	30	5	1,4,5,7,8	26	15–61	46	5	1,4,5,7,8
*T. araraticum*	30	23–59	36	1	5	24	19–31	12	1	5
*T. boeoticum*	51	24–92	68	3	1,2,5	54	22–177	155	3	1,2,5
*T. dicoccoides*	43	15–109	94	3	1,3,5	64	14–190	176	4	1,2,3,5
*T. dicoccon*	34	31–40	9	1	8	43	16–135	119	2	2,8
*T. durum*	31	10–50	40	3	1,5,8	23	14–50	36	3	1,5,8
*T. monococcum*	48	32–85	53	4	1,6,7,8	52	28–89	61	4	1,6,7,8

References: (1) Cakmak et al. 2000; (2) Monasterio and Graham, 2000; (3) Cakmak et al.^2004^; (4) Chhuneja et al.^2006^; (5) Rawat et al. 2009; (6) Ozkan et al. 2007; (7) Erba et al. 2011; (8) Zhao et al. 2009; (9) Arora et al. 2019

The same is true for *Aegilops* accessions, which have been investigated only sporadically, although some accessions could be a promising resource for biofortification. Compared to cultivated bread wheat, *Ae. tauschii* and *Ae.* *kotschyi* showed higher grain Fe and Zn concentrations (Chhuneja et al. 2006; Rawat et al. 2009) (Table 1).

Tetraploid wild emmer wheat (*T.* *dicoccoides)* has always received attention as this species provides a valuable source of genetic variation in terms of accumulation of micronutrients (Table 1) (Cakmak et al. 2000; Peleg et al. 2008). Higher accumulation of Fe and Zn in *T. dicoccoides* compared to *T.* *durum* was also consistent under water deficient conditions (Peleg et al. 2008). Compared to modern wheat cultivars, wild emmer wheat showed a higher variation in Zn and Fe concentration (Cakmak et al. 2004). Comparing the variation between Fe and Zn in wild diploid *T. boeoticum* and wild tetraploid emmer wheat, the amplitude for Zn was higher than for Fe (Cakmak et al. 2000).

Studies that analyzed iron and/or zinc in wild wheat accessions were summarized to calculate an overall mean (mg/kg) (Table 1). Further, the mean value of species analyzed in at least two independent studies was used for comparison between species. Due to the small number of studies, the median and the individual values were visualized (Fig. 4) (Weissgerber et al. 2015). *Aegilops* accessions showed higher iron accumulation compared to *Triticum* taxa across different studies (Fig. 4). However, the highest value for grain Fe concentration in *T.* *boeoticum* was in the same range as the median Fe concentration in *Ae. kotschyi* and *Ae.* *tauschii*. Such clear differentiation was not present for grain Zn concentration across species. The variation in grain Zn concentration was higher, as shown by the wider distribution of the mean values. This variation was pronounced in *T.* *dicoccoides*, which showed the highest median for grain Zn concentration (Fig. 4). This confirms the observations that the variation is higher for Zn accumulation than for Fe. It is noteworthy that this enrichment and variation found in wild emmer has not been passed on and confirmed in its economically important progeny *T. durum* (Fig. 4).

### Factors influencing the Fe and Zn concentration in the grain

How, if and to what extend the micronutrient concentration in the grain is influenced by external factors and the phenotype, e.g., grain size, is contradictory to date. Some authors argue that enhanced micronutrient density may come at the expense of agronomic performance. Progress in breeding for higher yields may have diluted the concentration of micronutrients in the grain, which is referred to as the dilution effect or reversible concentration effect in small grains (Cakmak et al. 2000). This hypothesis is supported by observations of negative correlations between yield and grain Zn concentrations (Oury et al. 2006). Negative correlation also occurred between the year of release of the respective variety and its micronutrient density (Zhao et al. 2009), suggesting that the breeding for high yield negatively affects micronutrient concentrations. This observation was more pronounced for Zn compared to Fe (Oury et al. 2006; Morgounov et al. 2007). On the other hand, some studies found no correlation between seed size and micronutrient concentration, calling into question the hypothesis of the dilution effect (Arora et al. 2019; Distelfeld et al. 2007). The micronutrient content per seed was analyzed in addition to the concentration. Seeds with the highest concentration also had the highest content per seed due to high seed weight and size (Cakmak et al. 2004, 2000). This clearly refutes the dilution hypothesis. In conclusion, the results concerning the relationship between agronomic performance and micronutrient density are controversial, suggesting that both components contribute to micronutrient density.

To identify the traits associated with grain micronutrient concentrations, correlations with other grain characteristics were analyzed. Agronomic management practices can help to increase micronutrient concentrations in the grain. Plants grown under nitrogen-sufficient conditions showed enriched micronutrients levels compared to nitrogen-deficient conditions (Monasterio and Graham 2000). Furthermore, higher grain protein content was also associated with higher micronutrients (Chatzav et al. 2010; Peleg et al. 2008). Fe and Zn are preferentially stored in the aleurone layer and the embryo. At the same time, the embryo contains high protein contents, which can explain the positive associations (Morgounov et al. 2007). As nutrients are translocated from the leaves to the grain during grain filling, nutrient concentrations (N, Fe, Zn) in the flag leaf were correlated with grain concentrations (Rawat et al. 2009). These findings suggest that grain protein is a sink for micronutrients and explain why bread wheat shows lower micronutrient concentration than durum wheat (Cakmak et al. 2010). In conclusion, an adequate supply of nitrogen can help to exploit the full potential of micronutrient accumulation in the grain. However, there is also another genetic component that supports wild wheats in the enrichment of micronutrients in the grain.

### Genetic properties of micronutrient accumulation in wheat wild relatives

Micronutrient uptake and translocation are quantitatively controlled and inherited traits (Arora et al. 2019) that can be genetically dissected by genome mapping. Therefore, Recombinant Inbred Lines (RIL) from two domesticated einkorn lines were developed by Ozkan et al. (2007) in order to gain insights into the genetics of micronutrient uptake. They found a promising Quantitative Trait Loci (QTL) on the short arm of chromosome 5 that contributes micronutrient accumulation (Ozkan et al. 2007). Chromosome 5A also harbored favorable QTLs for Fe and Zn micronutrient concentrations in a tetraploid RIL population derived from durum wheat and wild emmer. The authors stated that the wild alleles increased micronutrient accumulation (Peleg et al. 2009). Furthermore, chromosome 6B was detected as a promising gene source conferring high micronutrient concentrations in grains (Peleg et al. 2009; Cakmak et al. 2000, 2004). RIL lines containing chromosome 6B of *T.* *dicoccoides* had an enriched Zn concentration in the grain (Cakmak et al. 2000, 2004). Independent of this finding, the QTL *GPC-B1* on the short arm of chromosome 6B of *T.* *dicoccoides* was associated with elevated grain protein concentration (Joppa et al. 1997). *GPC-B1* showed a direct influence on chlorophyll degradation of flag leaves, thus accelerating senescence, and had pleiotropic effects on grain protein (Uauy et al. 2006a). Consequently, the transportation mechanism of nitrogen from leaves to grains was investigated for its role in grain micronutrient content. Recombinant chromosome substitution lines carrying the *GPC-B1* allele from wild emmer wheat showed increased Zn and Fe concentration in the grain compared to the *GPC-B1* allele from durum wheat (Distelfeld et al. 2007). The QTL was narrowed down to the allele *NAM-B1*, which encodes a NAC transcription factor (Uauy et al. 2006b). Reduced expression of *NAM-B1* resulted in plants with lower levels of protein, Zn and Fe in the grain, but higher levels in the flag leaf. This proved the role of *NAM-B1* in remobilization of micronutrients and nitrogen (Uauy et al. 2006b) and further explained the observed positive correlation between these components described above. Only wild wheat species possessed the functional *NAM-B1* genes (*GPC-B1* gene, used synonymously), leading to higher accumulation of micronutrients compared to *T. durum* and *T. aestivum*, where the allele is non-functional. Hexaploid wheat contains two functional homologous genes on chromosomes 6A and 6D (Uauy et al. 2006b). This genetic alteration occurred during wheat evolution, emphasizing the benefit of wild relatives. Therefore, the genomes of wheat relatives were explored for similar loci. Orthologues of *NAM-B1* were identified in *T. timopheevii*, *Ae. speltoides*, *Ae. bicornis*, *Ae. longissima*, *Ae. searsii*, and *Ae. sharonensis* (Hu 2012; Hu et al. 2013). Apart from some Single Nucleotide Polymorphisms (SNP), the *T.* *timopheevii NAM-G1* orthologue resembled the *T. dicoccoides NAM-B1* gene in promoting grain protein content and was therefore considered as a putative target for micronutrient enrichment. However, the follow-up study by Hu et al. (2017) showed no association between *NAM-G1* expression levels and grain Fe and Zn concentration. Consequently, they proposed a quantitatively controlled trait for micronutrient concentrations in grain rather than just *NAM-G1* as a single gene causing the higher micronutrient content (Hu et al. 2017). Another explanation for this finding could be that the allele does not function properly due to *T. timopheevii*’s domestication status. To confirm this, a study with its wild ancestor *T. araraticum* would be required. Additionally, *Ae. speltoides* or an extinct close relative of it participated as donors for the B and G genome. However, different genoytpes were involved in the independent polyploidization events of the emmer and timopheevii lineage. Since the functional *NAM* gene is found on the B1 genome, it might be non-functional in G1, thus explaining the lower micronutrient concentration in *T.* *timopheevii*.

### Regulatory role of the *GPC* gene

**Fig. 5:**
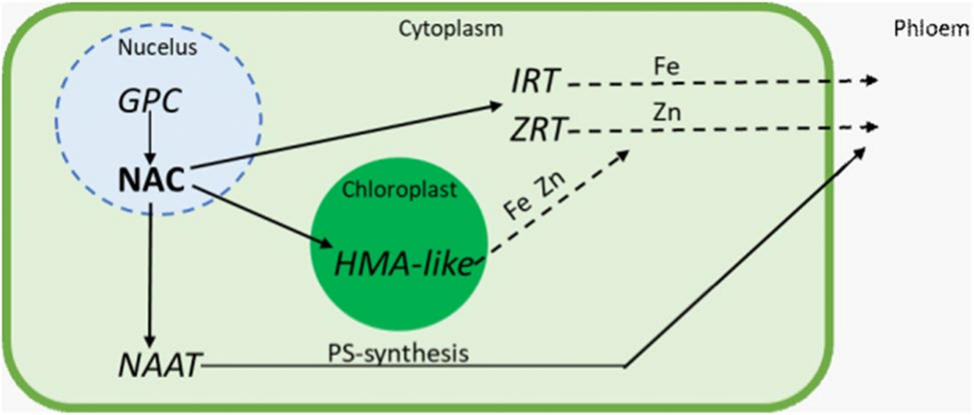
Regulatory role of the *GPC*-encoded NAC transcription factor for micronutrient translocation. Bold arrows indicate regulated transporters; dashed arrows show the transport of micronutrients. *GPC* = grain protein content gene; NAC = NAC transcription factor; *IRT* = iron-regulated transporter; *ZRT* = zinc-regulated transporter; *HMA-like* = Heavy Metal ATPase-like; *NAAT* = nicotianamine aminotransferase; PS = phytosiderophores

The physiological implications of this wild gene in hexaploid wheats were studied to elucidate its role in metal homeostasis. The pre- and post-anthesis phases are the most critical for micronutrient accumulation. Fe and Zn are not directly transferred into the grain. Initially, they are stored in the leaves, where they play an important role in the function of essential enzymes in mitochondria and plastids (Gupta et al. 2020). *GPC* is expressed during early senescence to prepare the plant for subsequent physiological processes (Cantu et al. 2011). Transcriptome analysis showed that under a high *GPC* expression levels, genes involved in transport, protein metabolism and catabolic/catalytic processes are also induced (Cantu et al. 2011). To explain the highly efficient translocation of micronutrients from flag leaf to grain enabled by *GPC*, Fe and Zn transporters were analyzed under putative *GPC* expression control. A Heavy Metal ATPase-like (HMA-like) was induced in the presence of *GPC* but was downregulated in *gpc* knock out mutants. HMAs and HMA-like transporters enable the transfer of micronutrients from the chloroplast to the cytoplasm. From here, the released nutrients must reach the phloem. Genes from the ZIP family, which include transporters for Fe (IRT = iron regulated transporter) and Zn (ZRT = zinc regulated transporter), formed the majority of upregulated genes under *GPC* influence. For phloem transport, Fe and Zn must be chelated. In this context, a gene involved in the biosynthesis of the phytosiderophores (PS) nicotianamine (NA), nicotianamine aminotransferase (NAAT), was found at a higher level (Pearce et al. 2014) (Fig. 5). In line with this finding, effective uptake and transportation via PS have also been suggested to be responsible for the higher micronutrient concentration in *Aegilops* species compared to *Triticum* species (Neelam et al. 2010). A genome-wide association study with the D genome donor *Ae. tauschii* confirmed the role of PSs by identifying marker–trait associations for iron concentrations. A significant locus was found near a gene involved in PS secretion (Arora et al. 2019). This shows that a subset of genes is controlled by the transcription factor encoding *GPC* gene, which facilitates the translocation of micronutrients. It is a unique example on how valuable wild alleles can be used for biofortification. However, since the *GPC* was not solely responsible for the accumulation of micronutrients in the grain, other genes need to be explored.

The identification of the functional *GPC* gene helps to understand the observed correlations between grain protein, yield and micronutrients explained above. First, it provides a plausible explanation for *T. dicoccoides* being such a promising source for higher grain micronutrient concentrations due to the functional *GPC-B1* allele. However, it remains unclear why there is such a high variation for Zn and Fe between *T.* *dicoccoides* accessions and how values as low as 15 mg/kg for Fe and 14 mg/kg for Zn can occur despite the presence of these functional alleles. One explanation could be a pronounced environmental influence, e.g., low micronutrient concentration in the soil. However, the wild *GPC-B1* alleles showed a stable contribution across different environments and no interaction between genotype and environment (Distelfeld et al. 2007). Either, these accessions lack the gene, perhaps due to adaption effects to different areas, or other genes associated with micronutrient accumulation may be equally important, which remain to be identified.

Second, the *GPC* gene might be involved in mechanisms leading to the dilution effect suggested earlier. Domesticated varieties contain functional orthologues of the *GPC* allele only on the A and D genomes, which are less efficient in micronutrient translocation from the flag leaf to the grain than the copy on the B genome. Since the *GPC*-mediated translocation is more efficient for Fe than for Zn (Waters et al. 2009; Avni et al. 2014), the lower grain Zn concentration in modern varieties can be explained. However, as stated above *T. dicoccoides* shows a high variation for micronutrients. Assuming that only selected *T. dicoccoides* genotypes were domesticated, there might be a chance that the chosen ones were low accumulators and this trait has been passed on to the domesticates. Regarding a negative effect on yield, the results are contradictory. In a study with recombinant chromosome substitution lines of *T. durum* and *T. dicoccoides*, the functional *GPC-B1* allele did not affect yield (Distelfeld et al. 2007), while in another study a functional allele in hexaploid wheat led to reduced grain weights due to accelerated senescence (Waters et al. 2009). In conclusion, the wild *GPC-B1* allele appears to have a differential effect on different genotypes. When introducing this allele into modern varieties, the genotype-specific yield responses need to be taken into account.

It would be interesting to see if there is an underling geographical pattern indicating environmental factors that have shaped genetic variation enabling biofortification. The bioavailability of micronutrients in cereal grains is limited by phytate, which binds Fe and Zn in insoluble forms and thus makes them unavailable for humans. To date, there is a lack of studies reporting phytate data from wheat wild relatives, which should be addressed in future research along with micronutrient analyses. Overall, the wheat genepool is still poorly explored in terms of diploid species and the timopheevii lineage. These species need to be considered in future research projects. Some of the transport and accumulation pathways of micronutrients in wheat have already been identified. The underlying translocation mechanisms can be targeted in wheat wild relatives to identify mechanisms that can be used in the breeding of biofortified wheat.

## Gluten in wheat wild relatives

Gluten is the main form of storage protein in the grain and determines the baking quality. Up to 90% of the protein in the seed are gluten. The gluten protein group includes two components, glutenins and gliadins, which differ in molecular weight and sulfur (S) content (Shewry 2019). Glutenins control dough strength and elasticity but make up only for 10% of the seed storage protein. These polymers are classified according to their molecular weight into low molecular weight glutenin subunit (LMW-GS) and high molecular weight glutenin subunit (HMW-GS). The latter is further subdivided into x and y HMW-GS. Gliadins are monomers comprising the S-rich prolamins α/β and γ gliadins and the S-poor ω-gliadins. Gliadins are responsible for the viscosity of the dough (Wieser 2007; Biesiekierski 2017; Shewry 2019). The gluten composition in the grain is strongly dependent on the genotype (Wieser 2007). Thus, different *Triticum* species show diversity for different quality and health properties.

### Wheat wild relatives harbor rare y-type high molecular weight glutenin subunits

Glutenins are important for the baking quality of the flour and form the backbone of the gluten network. Glutenin content increases in the order of diploid < tetraploid < hexaploid wheats (Geisslitz et al. 2019). The *Glu1* loci on the long arm of chromosome 1 homologous encode two HMW-GS genes, the x- and y-type subunits (Li et al. 2020b; Payne et al. 1980, 1984). The former has a higher molecular weight than the latter. HMW-GS that originate from the D genome influence dough properties the most (Li et al. 2020b). However, y-type subunits are also important for dough quality, although the y-subunit of the A genome (1Ay) is usually not expressed in hexaploid bread wheat (Waines and Payne 1987). Due to the higher number in cysteine residues compared to the x-subunit, the y-subunit is a valuable source for increasing dough quality (Li et al. 2020b).

The variation in HMW-GS decreased with increasing ploidy status during evolution (Xu et al. 2009). The diploid wheat species showed the highest variation in molecules encoding the 1Ay subunit (Hu et al. 2012). The diploid A genome donor of wheat *T.* *urartu* contained different 1Ay subunit variants (Xu et al. 2009; Caballero et al. 2008; Hu et al. 2010). However, not all 1Ay subunits were expressed (Xu et al. 2009). This pattern was especially found in *T. urartu* accessions from Turkey, Armenia, Iraq and Iran (Waines and Payne 1987). Yet, the variation for 1Ay subunits was characterized by a rare allelic distribution (Caballero et al. 2008). Prediction of the secondary structure of the 1Ay subunit of *T. urartu* showed a potentially beneficial structure for flour processing quality (Hu et al. 2010). *T. boeoticum* may be another source of 1Ay subunit introduction (Waines and Payne 1987). The 1Ay subunit encoded by this species was different from the expressed 1Ay subunit of *T. urartu* (Hu et al. 2012; Jiang et al. 2009). The 1Ay subunit of *T. boeoticum* had a higher molecular mass and was thus longer, which favors the formation of an enhanced gluten network due to interchain bonds (Jiang et al. 2009). Introgression of the *Glu-A1* locus of *T. boeoticum* into bread wheat resulted in an improved dough quality (Rogers et al. 1997). The domesticated einkorn *T. monococcum* contained functional 1Ay subunits, two of which were investigated for their molecular characterization (Guo et al. 2013). One allelic variant showed an additional cysteine residue, which could improve dough quality (Guo et al. 2013). The diversity of 1Ay subunits was higher in *T. boeoticum* and *T. monococcum* than in *T. urartu* (Hu et al. 2012). Thus, the diploid wheats contain useful diversity for enriching dough quality, which should be exploited.

*T. dicoccoides* contained the highest variation in 1Ay subunits among the tetraploid wheat species (Hu et al. 2012). An active 1Ay subunit in *T. dicoccoides* (GenBank accession: JF519636) resembled the pattern of amino acid sequences of the 1Ay gene found in *T. urartu*, but they differed in their predicted secondary protein structure (Bi et al. 2014). However, in a phylogenic analysis, this 1Ay allele of *T. dicoccoides* clustered together with another *T. dicoccoides* 1Ay subunit (GenBank accession: KC545956). This in turn was introgressed into hexaploid wheat and positively associated with improved dough quality (Wang et al. 2018). The former subunit allele (JF519636) and probably other active 1Ay alleles of *T. dicoccoides* thus harbor putative beneficial quality traits.

The wild tetraploid counterpart *T. araraticum* in the GGAA lineage showed only three different 1Ay subunits (Hu et al. 2012). Two of these 1Ay subunits were similar to *T. boeoticum,* and three were similar to *T. monococccum* (Hu et al. 2012). In *T. timopheevii*, only one 1Ay subunit was found that matched a subunit from *T. monococcum* (Wan et al. 2002; Hu et al. 2012). This variant also occurred in *T. zhukovskyi*. The other variant of the hexaploid GGA^u^A^u^A^m^A^m^ *T. zhukovskyi* taxon did not occur in one of its putative ancestors (Hu et al. 2012). The variant 1Ay8 subunit in *T. monococcum* was favorable for bread making quality (Guo et al. 2013). *T. araraticum* contained the homologous 1By8 subunit (Hu et al. 2012), which could make *T. araraticum* a potential source for quality improvement.

Wheat wild relatives show valuable y-type HMW-GS that promise improved dough quality. In particular, all diploid ancestors harbor favorable variants. The less explored GGAA lineage and especially *T. zhukovskyi* may be a valuable source for y-type subunits due to its kinship with both diploid A-genome wheat species, *T. urartu* and *T. monococcum*. This is also an advantage over the BBAA and BBAADD wheat species. As the diversity of the active 1Ay subunits decreased during wheat evolution and domestication history, it is of great importance to conserve the wild relatives.

### Alpha-gliadins in wild wheat—a safe option for celiac disease patients?

The monomeric gliadins comprise a large gene family with high diversity due to their long evolutionary history. The *Gli* locus comprises genes that encode for gliadins. ω and γ gliadins are encoded by the *Gli1* locus on chromosome 1 homologous. The short arm of chromosome 6 homologues contains the *Gli2* locus, which encodes α/β gliadins (Payne 1986). In addition to its quality-determining properties, gluten has a negative effect on patients with celiac disease (CD). The primary trigger for CD are α-gliadins, whereas the other gliadin groups appear to be less harmful but not entirely safe for some CD patients (Biesiekierski 2017). In the small intestine, gluten is digested (deamidated) and epitopes are formed during this process. These epitopes confer a T cell response that is activated by *HLA-DQ2* and *-DQ8* (HLA = Human leukocyte antigen) and leads to an autoimmune response (reviewed in: Biesiekierski 2017). Alpha-gliadins containing the 33mer peptide are most likely to cause CD. The 33mer peptide comprises the epitopes DQ2.5-glia-α1a and b, DQ2.5-glia-α2 and DQ2.5-glia-α3. The immunodominant 33mer fragment originates from the D genome (Schaart et al. 2020). Therefore, diploid and tetraploid wheats might confer a less toxic immune response.

Domesticated einkorn *T.* *monococcum* contained more gliadin and thus more gluten than common wheat. This observation was consistent across different locations, leading to the assumption that the higher gluten and gliadin are a characteristic of einkorn wheat (Geisslitz et al. 2019). It is noteworthy that *T. boeoticum*, the wild ancestor of *T.* *monococcum*, showed higher gliadin content compared to its domesticate (Ozuna and Barro 2018). However, at the molecular level, *T. urartu* and *T.* *monococcum* lacked some of the toxic epitopes and thus the 33mer peptide (Molberg et al. 2005; Salentijn et al. 2009; Zhang et al. 2015; van Herpen et al. 2006; Ozuna and Barro 2018). Spaenij-Dekking et al. (2005) also found less alpha-gliadins in diploid A genomes. A comparative toxicity study suggested that gliadin of *T. monococcum* would be safer for CD patients than gliadin from *T.* *aestivum* (Pizzuti et al. 2006). However, *T. monococcum* harbors many different, putatively immunogenic and toxic epitopes; thus, it cannot be considered a CD-safe food only because of the absence of a 33mer peptide (Vaccino et al. 2009; Ozuna et al. 2015). The same is true for *T.* *urartu*, which lacks the DQ2.5-glia-α2 epitope, but contains DQ2.5-glia-α1a and DQ2.5-glia-α3 CD-triggering epitopes (Zhang et al. 2015; Ruiz-Carnicer et al. 2019). This implies that regarding the quantity and molecular composition of gluten, einkorn is not a safe option for CD patients.

Compared to einkorn, emmer wheat contained less gliadin and gluten, but more than hexaploid bread wheat (Geisslitz et al. 2019). The potential B/G genome donor *Ae.* *speltoides* also lacked the 33mer peptide (Molberg et al. 2005; Schalk et al. 2017), and conclusively, some of the *T. dicoccon* and *T. durum* samples showed the same phenotype (Molberg et al. 2005). The B genome in tetraploid and hexaploid wheat showed the lowest expression level compared to *Gli A2* and *Gli* *D2* (Salentijn et al. 2009). Further, it harbored the lowest amount of toxic epitopes (Ozuna et al. 2015). Indeed*, Ae.* *speltoides*, *Ae.* *longissima* and *Ae. searsii* showed no abundance of any of the canonical epitopes (Ruiz-Carnicer et al. 2019). However, genes encoding for α-gliadins in *T. dicoccoides* encode toxic epitopes and thus constitute a threat for CD patients (Qi et al. 2006, van den Broeck et al. 2010, Ozuna and Barro 2018). However, the CD-triggering α-gliadin epitopes are most likely retrieved from the A genome (van den Broeck et al. 2010). Alpha-gliadins from the A genome in *T.* *dicoccoides* contained the T cell epitopes DQ2.5-glia-α1a and DQ2.5-glia-α3, which resembles the pattern in *T. urartu* (Huo et al. 2019; Zhang et al. 2015). In summary, the B genome of tetraploid wheats can be considered the least toxic, but due to the presence of the A genome, tetraploid wheats remain unsuitable for CD patients. However, the content of toxic epitopes varied in tetraploid wheat and decreased as domestication progressed (van den Broeck et al. 2010; Ozuna and Barro 2018). Thus, domesticated species seem to be more suitable for breeding wheat with fewer CD-triggering epitopes. It has to be kept in mind that this approach will generally only help a part of the CD patients, as the variability of toxic epitopes is high and the response to these depends on the individual.

*Ae. tauschii* formed all of the three harmful epitopes. The DQ2.5-glia-α2 and DQ2.5-glia-α1b epitopes were exclusively assigned to the D genome and were only found in hexaploid wheat, suggesting that this epitope was inherited via the D genome donor (Ruiz-Carnicer et al. 2019; Ozuna and Barro 2018). *Ae. tauschii* showed a high diversity for unique α-gliadin peptide variants, but only accessions from the potential geographic origin of hexaploid wheat hybridization (south-west Caspian Sea) contained the toxic 33mer peptide. On the one hand, this suggests that the CD-triggering epitope in bread wheat originates from this geographic region, but on the other hand, it also underlines that there are many *Ae. tauschii* accessions which might contain less to no toxic peptide variants (Schaart et al. 2020).

The 33mer peptide is the main trigger of CD; however, it seems that even partial epitopes can already cause a reaction in CD patients. Regarding the abundance of canonical epitopes, the D genome donor seems to show the highest profusion, followed by BAD and the A genome donor. The B genome is the least harmful in terms of toxic epitopes (Ruiz-Carnicer et al. 2019). It is still unclear whether the G genome has less toxic α-gliadins to offer, but the timopheevii lineage does not seem to be a promising resource for low α-gliadin epitopes, as the tetraploid *T. araraticum* and *T. timopheevii* potentially have the same A genome donor species as the emmer lineage. Moreover, *T.* *zhukovskyi* is probably even worse because the A genomes of *T. urartu* and *T. monococcum* are present. Nevertheless, one has to consider that the most toxic 33mer peptide comes from the D genome, which is absent in the timopheevii lineage. Therefore, it might be worthwhile to identify other epitopes there.

## Phenolic acids in the wheat genepool

Phenolics occur in free, soluble conjugated and insoluble bound forms, with the latter form being the most abundant (Adom and Liu 2002). The differently bound phenolic acids differ in their bioavailability (Laddomada et al. 2015). Ferulic acid is the major phenolic acid found in wheat (Hernández et al. 2011), and it follows the pattern of the free, soluble conjugated and insoluble bound phenolics at a ratio of 0.1:1:100 (Adom and Liu 2002). Wheat relatives have been investigated for their content in phenolic acids due to their antioxidant capacity.

Data on the phenolics content of wild wheats are rare and contradictory. The wild diploid wheats *T. urartu* and *T. boeoticum* showed higher total polyphenol content (TPC) compared to the domesticated *T. monococcum*, *T. dicoccon* and *T. aestivum*, in which TPCs were almost the same (Brandolini et al. 2015; Yilmaz et al. 2015), while *T. durum* grouped in between (Brandolini et al. 2015). The wild tetraploid emmer revealed the lowest phenolic content among domesticated emmer, *T. durum* and other tetraploid emmer landraces, thus implying inferiority of phenolic content of the wild ancestor of the tetraploid wheats (Laddomada et al. 2017). Some data are available for the domesticated einkorn and emmer. Emmer showed a higher total phenolic content compared to einkorn, which was even lower than the total phenolic content of bread wheat (Serpen et al. 2008). This pattern was also found in the ferulic acid content (Serpen et al. 2008). The opposite was reported by Barański et al. (2020), who measured significantly higher total phenolic acids in einkorn compared to emmer. However, this study only contained one einkorn line (Barański et al. 2020). Both studies revealed contradictory result compared to the equal phenolic content of emmer and einkorn reported by Brandolini et al. (2015). A summary of studies analyzing *T. monococcum*, *T. dicoccon*, *T. durum* and *T. aestivum* with the same method and an overall comparison of studies with different methods were compiled by Shewry and Hey (2015). In the former comparison, einkorn ranked last in the total phenolic acid and ferulic acid content and emmer ranked first, also showing a high variation. In their comparison of different studies, the authors found no distinct differences in the phenolics content across species due to high variability (Shewry and Hey 2015).

In conclusion, no clear pattern can be identified from those studies. This might also be due to different analytical methods applied (Shewry and Hey 2015). Hence, there is no clear evidence that wild wheats possess valuable characteristics for the improvement in phenolic contents in wheat.

## The grain quality in an evolutionary context

Evolution describes the gradual process of change and development in populations over time. Thereby, new characteristics or traits develop or disappear. The discussed grain quality characteristics micronutrients and gluten indicate an evolutionary pattern in their variation and accumulation. The main drivers for this phenomenon were a transition from wild to domesticated species and the change in ploidy levels. First of all, the variation for Fe/Zn and 1Ay-glutenin subunits was higher in wild relatives of wheat compared to the domesticated species (Table 1, Fig. 4) (Cakmak et al. 2000, 2004; Peleg et al. 2008; Hu et al. 2012). This implies that during the transformation from wild to cultivated species, those traits were unintentionally altered and fixed, thus reducing the resulting diversity. However, the process of domestication also contributed to more stable grain quality traits, which is especially important for the baking quality. Besides the variation, the level of accumulation of Fe and Zn and α-gliadins also showed an evolutionary trend. The domesticated species harbored lower accumulation potential than the wild relatives. In the case of micronutrients, the gene *GPC-B1*, which turned non-functional during emmer domestication, underlies this phenomenon (Uauy et al. 2006b). High variation and accumulation potential for these grain constituents are thus features of wheat wild relatives that could be exploited in wheat improvement. In the case of phenolics, neither a clear pattern for changes in variation nor for an enrichment of phenolic content in wild and domesticated wheats was identified due to the lack of studies. However, it seems that there might be no evolutionary pattern for this secondary metabolite. Another evolutionary phenomenon that may have affected grain quality is allopolyploidization. The change in ploidy status caused different patterns for gliadin and glutenin during evolution: while an increase in ploidy reduced the gliadin content, the opposite was true for glutenin (Geisslitz et al. 2019).

Due to a lack of information in the scientific literature, the neglected timopheevii lineage should be analyzed regarding its grain quality. As with other wild species, the wild *T. araraticum* would be expected to exhibit high variations in Fe and Zn. Similar α-gliadin derived epitopes were found in *T. urartu* and *T. dicoccoides* and were both obtained from the A genome. Those epitopes would be likely to occur in the timopheevii lineage due to its ancestry with *T. urartu,* and furthermore, *T. zhukovskyi* might additionally contain the epitopes from *T. monococcum*. However, the toxic 33mer peptide is less likely to be represented in this lineage because of the missing hybridization with *Ae. tauschii*. Wild *T. araraticum* could also be a potential source for variation for glutenin subunits, but higher glutenin concentration would be expected in the domesticated tetra- and hexaploid species due to their polyploidization status. These assumptions need scientific evidence, but if they hold true, they could support the concept that evolutionary history can help to identify beneficial species.

## Conclusion

The wild relatives of wheat harbor a large genetic diversity for certain grain quality traits such as minerals or gluten content. Additional important grain quality traits such as starch composition, fatty acid composition and non-starch polysaccharide concentration in wheat wild relatives have barely been investigated in the past. Thus, exploring the variation in these compounds in the wild relatives could be interesting topics for future studies. So far, the focus of most quality traits research has been on *T.* *dicoccoides* because of its straightforward use in bread wheat improvement. Identification of the underlying causative loci and polymorphism can be difficult in wild relatives due to the lack of reference genome sequences. However, ongoing advances in sequencing technology can simplify the development of reference genomes sequences for wheat wild relatives (Avni et al. 2017; Maccaferri et al. 2019; Ling et al. 2018; Walkowiak et al. 2020; Luo et al. 2017; Pont et al. 2019). In addition, the development of a wheat pan-genome sequence can be a valuable strategy in harnessing the genetic diversity of wheat wild relatives (Khan et al. 2020). Furthermore, the advent of genome editing enables de novo domestication strategies for targeted use of crop wild relatives (Fernie and Yan 2019; Zsögön et al. 2017, 2018; Xie and Liu 2021). However, ex situ conservation in genebanks constitutes an important strategy for safeguarding this unexplored diversity and also provides useful information/passport data of the accessions (https://www.genesys-pgr.org). In this context, the diploid taxa should receive more attention, due to their potential for enhancing grain micronutrient concentration as well as y-type HMW-GS. The same applies to the neglected GGAA lineage of wheat, in which quality traits remain to be investigated. In conclusion, further exploring the extended wheat gene pool harbors great potential for wheat diversification and quality improvement.

**Online Resource Map:** https://de.m.wikipedia.org/wiki/Datei:A_large_blank_world_map_with_oceans_marked_in_blue.PNG.
